# Comparison of a high and a low intensity smoking cessation intervention in a dentistry setting in Sweden – a randomized trial

**DOI:** 10.1186/1471-2458-9-121

**Published:** 2009-04-30

**Authors:** Eva Nohlert, Åke Tegelberg, Per Tillgren, Pia Johansson, Andreas Rosenblad, Ásgeir R Helgason

**Affiliations:** 1 Centre for Clinical Research, Uppsala University, Central Hospital, Västerås, Sweden; 2 Faculty of Odontology, Malmö University, Malmö, Sweden; 3 Department of Public Health Sciences, Karolinska Institutet, Stockholm & School of Health, Care and Social Welfare, Mälardalen University, Västerås, Sweden; 4 Unit of Health Economics, Stockholm Centre of Public Health, Stockholm County Council & Department of Public Health Sciences, Karolinska Institutet, Stockholm, Sweden; 5 Departments of Oncology - Pathology and Public Health Sciences, Karolinska Institutet, Stockholm, Sweden & School of Health and Education, Reykjavik University, Iceland

## Abstract

**Background:**

Tobacco is still the number one life style risk factor for ill health and premature death and also one of the major contributors to oral problems and diseases. Dentistry may be a potential setting for several aspects of clinical public health interventions and there is a growing interest in several countries to develop tobacco cessation support in dentistry setting. The aim of the present study was to assess the relative effectiveness of a high intensity intervention compared with a low intensity intervention for smoking cessation support in a dental clinic setting.

**Methods:**

300 smokers attending dental or general health care were randomly assigned to two arms and referred to the local dental clinic for smoking cessation support. One arm received support with low intensity treatment (LIT), whereas the other group was assigned to high intensity treatment (HIT) support. The main outcome measures included self-reported point prevalence and continuous abstinence (≥ 183 days) at the 12-month follow-up.

**Results:**

Follow-up questionnaires were returned from 86% of the participants. People in the HIT-arm were twice as likely to report continuous abstinence compared with the LIT-arm (18% vs. 9%, p = 0.02). There was a difference (not significant) between the arms in point prevalence abstinence in favour of the HIT-protocol (23% vs. 16%). However, point prevalence cessation rates in the LIT-arm reporting additional support were relatively high (23%) compared with available data assessing abstinence in smokers trying to quit without professional support.

**Conclusion:**

Screening for willingness to quit smoking within the health care system and offering smoking cessation support within dentistry may be an effective model for smoking cessation support in Sweden. The LIT approach is less expensive and time consuming and may be appropriate as a first treatment option, but should be integrated with other forms of available support in the community. The more extensive and expensive HIT-protocol should be offered to those who are unable to quit with the LIT approach in combination with other support.

**Trial Registration:**

Trial registration number: NCT00670514

## Background

Even though Sweden is one of the leading high income countries in reducing the proportion of smokers in the population, tobacco is still the number one life style risk factor for ill health and premature death in Sweden [1]. Globally, tobacco-attributable deaths are projected to rise from 5.4 million in 2005 to 6.4 million in 2015 [2].

In many countries, dentistry may be a potential setting for several aspects of clinical public health interventions because of their regular recall system of patients, and thereby opportunity of assisting people to life style changes. There is a growing interest in several countries to develop tobacco cessation support in dentistry setting [3-5]. Tobacco use is one of the major contributors to oral problems and diseases in Sweden [6], including oral cancer, oral mucosal lesions and periodontal diseases. Also, tobacco use is a significant prognostic variable for dental implant survival [7-10]. That is probably why many Swedish dentists and dental hygienists regard smoking cessation support as a natural part of their work and have ambitions to develop tobacco cessation support at their clinics [5,11]. However, factors such as effectiveness, expenses and time are of central importance for the implementation of different kinds of smoking cessation interventions [12].

The aim of the present study was to assess the relative effectiveness of a high intensity intervention compared with a low intensity intervention, using the local dentistry as a setting for cessation support.

## Methods

The Swedish county of Västmanland, with 250 000 inhabitants, is a mixture of urban and rural areas. Västerås, the largest city, has approximately 134 000 inhabitants. During a period of 18 months (August 2003 through February 2005) dental and health care personnel, as well as industrial health service, in Västerås with surroundings were encouraged to screen for daily smokers and offer all smokers over 20 years of age smoking cessation support. People reporting to be both smokers and users of other tobacco products (combined users) were not excluded. Those accepting support were referred to the study administrator, for possible inclusion in a study. Excluded were people with reading difficulties and those not fluent in the Swedish language. Smokers meeting the inclusion criteria were randomly assigned to two study arms. One arm received support of relatively low treatment intensity, whereas the other group was assigned to high treatment intensity support. Power calculation estimated that 150 smokers would have to be recruited to each arm to statistically confirm a 5% difference between the groups. The randomization was performed by an independent person using an envelope technique in blocks of four [13].

After written consent had been obtained, a baseline questionnaire and a covering letter were mailed to the participants. They were requested to complete the questionnaire at home and bring it to the first appointment with the counsellor at the local dental clinic where they had been assigned. A follow-up questionnaire was sent by mail 12 months after the planned smoking cessation date along with a pre-stamped return envelope.

The study was approved by the ethical committee at Uppsala University (Dnr:Ups 02-457).

### Treatment protocols

All counselling was carried out by three dental hygienists who had been educated and trained in smoking cessation support methods in general and especially for the specific programmes used in this study. They were calibrated before the intervention programmes started.

The *High Intensity Treatment *program (HIT) comprised eight 40-minute individual sessions at the local dental clinic over a period of 4 months. The program was a traditional state of art smoking cessation program based on a mixture of behaviour therapy, coaching, and pharmacological advises. The program was based on a group session program previously used by the Public Dental Health Service in Västmanland and adapted for individual support [14].

The *Low Intensity Treatment *program (LIT) consisted of one 30-minute counselling session focusing on explaining the content of a traditional self-help program (in Swedish "Fimpa dig fri"). The leaflet contained an 8-week program with instructions and tasks to perform each week [14]. The self-help program included several tests and behaviour registration exercises suggesting different action plans for different outcomes. In general, the self-help program and the clinic-based program were based on similar treatment protocols. Information on possible benefits of Nicotine Replacement Therapy (NRT) was included in both programs, but the participants got no recommendation regarding use or not.

At the first meeting, a smoking cessation date was fixed for all participants in both groups. The participants were informed that they would be followed-up through a questionnaire after 12 months counting from their fixed smoking cessation date. Both programs were free of charge.

### The questionnaire and outcome measures

The questions used in the study were developed and tested for face validity by means of in-depth interviews and focus groups at the Centre for Tobacco Prevention in Stockholm [15] and had previously been used to assess the treatment effectiveness and cost effectiveness of the Swedish National Tobacco Quitline ("Sluta-röka-linjen") [16-18].

*Abstinence *was assessed with the question: Have you smoked during the past seven days? The answer alternatives were: No I have not smoked at all; Yes but not daily; Yes daily. Those who answered that they had not smoked at all during the past seven days answered an additional question, assessing the number of days they had been completely smoke-free from the time of follow-up and backwards.

*Point prevalence abstinence *was defined as self reported; "not one puff of smoke during the past seven days." *Continuous abstinence *was defined as self reported; "not one puff of smoke during the past six months (183 days)". The continuous abstinence is based on the cut-off for the maintenance stage according to the transtheoretical model of stages of change [19].

To assess the *intention to quit *we used the question: If you are still smoking, what are your intentions concerning trying to quit? The answer alternatives, based on the stages of change model [19], were: I am trying to quit just now; I intend to try to quit within one month; I intend to try to quit within the next six months; I do not intend to quit.

The question assessing *depressive mood *was: Have you been in a, for you, abnormally depressive mood during some period, since your first contact with the smoking cessation program? The answer alternatives were Yes and No.

The question about *other support *was: Is there anyone else besides The Public Dental Health Service in Västmanland who has supported you in your attempt to quit during the study period? The answers were categorized into Yes or No.

HIT-participants completing the program as well as all LIT-participants, received a more comprehensive follow-up questionnaire including the three abstinence and intent to quit questions, as well as questions about depressive mood, use of smokeless tobacco, other support and additional factors relevant for the study. Participants in the HIT-group not completing the program received a short questionnaire at 12-months follow-up comprising the main outcome measures; point prevalence and continuous abstinence at the time of follow-up, and a question on future plans to quit.

### Statistics

We used the "intention to treat" approach where all participants were included in the analyses according to the program they were randomized to. When measuring abstinence at follow-up, non-responders to the follow-up questionnaire were treated as smokers. For six persons who did not reply to the baseline questionnaire, we only have information about which program they were randomized to, and thus they could not be used in the analyses of background variables.

Chi-square test, Fisher's exact test and Mann-Whitney U-test were used for comparisons between HIT and LIT as well as between men and women for different independent variables. Logistic regression analysis was performed to calculate odds ratios (ORs) with 95% confidence interval for the outcome measures. The ORs express a risk or a chance of an event or state occurring and the reference value ("ref." in tables) is 1.0. First we made a univariate analysis of separate independent variables and then a multiple analysis including the same variables plus sex and age to get controlled ORs. Tests of homogeneity of the ORs from the univariate analyses were performed using Breslow-Day tests.

The statistical analyses were performed using the Statistical Package for Social Sciences (SPSS, version 14.0). Statistical significance was set to p < 0.05.

## Results

**Figure 1:**
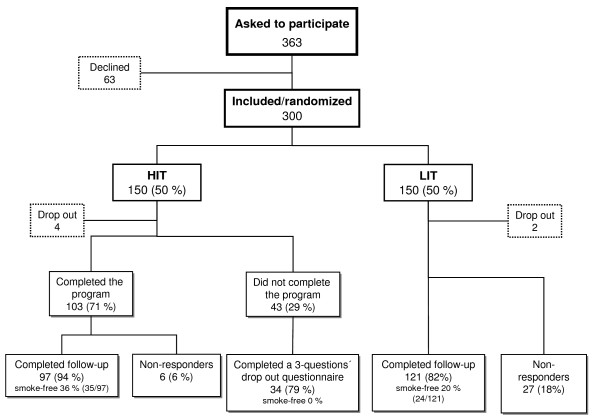
**Flowchart of the study**. Also presenting the proportion of people reported to be smoke-free (point prevalence) at the 12-month follow-up.

Of the 363 persons originally accepting to participate in the study, 63 changed their mind leaving 300 for study registration and randomization to the two arms. Of these, six did not reply to the baseline questionnaire, leaving 294 in the study population with complete information, 146 in the "High Intensity Treatment" (HIT) arm and 148 in the "Low Intensity Treatment" (LIT) arm (Figure 1). The dentistry recruited 143/294 (49%), the regional general health service 43/294 (15%), the local industrial health service 11/294 (4%), and 92/294 (31%) were recruited by direct advertisement in the local media. For 5/294 (2%) information was missing.

Forty-three participants in the HIT-group (29%) did not complete the program. Most of them left the program within the first three weeks and the most common reasons were personal problems, stress, lack of motivation, withdrawal symptoms and medical or psychological problems.

Answers on point prevalence and continuous abstinence were received from 252/294 (86%) of the participants at the 12-month follow-up, 131/146 (90%) responded to these questions in the HIT-group and 121/148 (82%) in the LIT-group (Figure 1).

At baseline there were no significant differences between the arms regarding assessed background variables. Approximately eight out of ten were women and the majority of the participants had 10 years or more of education (Tables 1 and 2). The participants in both groups had, on average, been daily smokers for 29 years, smoking a mean of 15 cigarettes per day in the week prior to the first interview (not in table). A similar distribution according to sex (75% women) was seen among the 63 persons who were invited but who declined participation before start.

**Table 1:** Population characteristics, % (n)

	**Total** (N = 294)	**High intensity treatment** (n = 146)	**Low intensity treatment** (n = 148)	**p-value**^†^
Recruited*				
**Age:**				
18 – 34	10 (29)	6 (8)	14 (21)	
35 – 49	41 (121)	47 (68)	36 (53)	.050
50 – 64	44 (128)	42 (62)	45 (66)	
65 – 84	5 (16)	6 (8)	5 (8)	

**Education in years:**				
0 – 9	23 (67)	21 (30)	25 (37)	.363
≥ 10	77 (227)	79 (116)	75 (111)	

**Smoker at first interview:**				
Yes	99 (291)	99 (144)	99 (147)	.621
No	1 (3)	1 (2)	1 (1)	

**Number of smoked cigarettes:**				
≥ 20/day	34 (99)	31 (45)	36 (54)	
10–19/day	51 (151)	52 (76)	51 (75)	.443
0–9/day	15 (44)	17 (25)	13 (19)	

* Recruited = all those who came to the first interview

^† ^Statistical significant difference between HIT and LIT tested with chi-square and Fisher's Exact test

**Table 2:** Population characteristics, % (n), division by sex

	**Total** (N = 294)			**High intensity treatment** (n = 146)			**Low intensity treatment** (n = 148)		
	
	**Men**	**Women**	**p-value**^†^	**Men**	**Women**	**p-value**^†^	**Men**	**Women**	**p-value**^†^
Recruited*	22 (64)	78 (230)	< .001	21 (30)	79 (116)	< .001	23 (34)	77 (114)	< .001

**Age:**									
18 – 34	8 (5)	10 (24)		7 (2)	5 (6)		9 (3)	16 (18)	
35 – 49	36 (23)	43 (98)	.139	33 (10)	50 (58)	.330^b^	38 (13)	35 (40)	.223^c^
50 – 64	45 (29)	43 (99)		50 (15)	41 (47)		41 (14)	46 (52)	
65 – 84	11 (7)	4 (9)		10 (3)	4 (5)		12 (4)	4 (4)	

**Education in years:**									
0 – 9	31 (20)	20 (47)	.068	33 (10)	17 (20)	.052	29 (10)	24 (27)	.498
≥ 10	69 (44)	80 (183)		67 (20)	83 (96)		70 (24)	77 (87)	

**Smoker at first interview:**									
Yes	98 (63)	99 (228)	.523	97 (29)	99 (115)	.370	100 (34)	99 (113)	1.000
No	2 (1)	1 (2)		3 (1)	1 (1)			1 (1)	

**Number of smoked cigarettes:**									
≥ 20/day	48 (31)	30 (68)		50 (15)	26 (30)		47 (16)	33 (38)	
10–19/day	34 (22)	56 (129)	.006	30 (9)	58 (67)	.016	38 (13)	54 (62)	.244^d^
0–9/day	17 (11)	14 (33)		20 (6)	16 (19)		15 (5)	12 (14)	

* Recruited = all those who came to the first interview

^† ^Statistical significant difference between men and women tested with chi-square and Fisher's Exact test

^a ^1 cell (12.5%) have expected count less than 5. The minimum expected count is 3.48

^b ^2 cells (25.0%) have expected count less than 5. The minimum expected count is 1.64

^c ^2 cells (25.0%) have expected count less than 5. The minimum expected count is 1.84

^d ^1 cell (16.7%) have expected count less than 5. The minimum expected count is 4.36

People in the HIT-group were twice as likely to report continuous abstinence compared with the LIT-group when analyzing the data using the "intention to treat" method and treating all non-responders as smokers. The results were almost identical when only those who had answered the follow-up questions were included in the analysis (Table 3). There was a small (not significant) difference between the arms in point prevalence abstinence in favour of the HIT-protocol (Table 3).

**Table 3:** Point prevalence and continuous abstinence at the 12-month follow-up, by treatment intensity.

	Point prevalence abstinence*			Continuous abstinence*		
	
	% (n/N)	p-value	OR (95% CI)	% (n/N)	p-value	OR (95% CI)
**Intention to treat approach – all non-responders treated as smokers:**						
**LIT **(ref.)	16 (24/150)	.11	1.0	9 (13/150)	.02	1.0
**HIT**	23 (35/150)		1.6 (0.9–2.8)	18 (27/150)		2.3 (1.1–4.7)

**Comparing people who answered the follow-up questions on abstinence:**						
**LIT **(ref.)	20 (24/121)	.20	1.0	11 (13/121)	.03	1.0
**HIT**	27 (35/131)		1.5 (0.8–2.7)	21 (27/131)		2.2 (1.1–4.4)

First analyzed as "intention to treat" then comparing those answering the follow-up questions on abstinence.

* Point prevalence abstinence = not smoked at all in the seven days prior to follow-up. Continuous abstinence = not smoked at all in the 6 months (≥ 183 days) prior to follow-up.

Comparing quit rates between men and women showed that the HIT method tended to be more efficient for both sexes, although the difference was significant only for the ORs among females for continuous abstinence. However, the difference in ORs between sexes was not statistically significant. Regardless of education level, the HIT protocol tended to be more efficient than the LIT protocol, although it was statistically significant only for continuous abstinence among people with a higher level of education. Furthermore, the quit rates tended to be higher among people with a higher level of education in both the HIT and the LIT program, but people with a lower level of education appeared to gain more from the HIT protocol, in that the ORs for HIT were higher in that group than in the group with higher education. However, these differences were not statistically significant.

Regarding intensity of smoking, the HIT protocol tended to be more efficient for all levels of intensity, although it was statistically significant only for continuous abstinence among people who smoked ≥ 20 cigarettes/day. Further, the fewer cigarettes smoked a day, the higher were the quit rates in both the HIT and the LIT program, but people who smoked ≥ 20 cigarettes/day appeared to gain more from the HIT-protocol, since the ORs for HIT were higher among them than among people who smoked fewer cigarettes. These differences were, however, not statistically significant (Table 4).

**Table 4:** Abstinence (point prevalence and continuous) by sex, education level and number of smoked cigarettes at baseline, as well as odds ratios for the two abstinence standards, using all randomized subjects.

	**Point prevalence abstinence**^†^		**Continuous abstinence**^†^	
	% (n/N)	OR (95% CI)	% (n/N)	OR (95% CI)

**Education**: 0–9 years				
LIT (ref.)	8 (3/37)	1.0	5 (2/37)	1.0
HIT	20 (6/30)	2.8 (0.6–12.5)	13 (4/30)	2.7 (0.5–15.8)
**Education**: ≥ 10 years				
LIT (ref.)	19 (21/111)	1.0	10 (11/111)	1.0
HIT	25 (29/116)	1.4 (0.8–2.7)	20 (23/116) *	2.2 (1.04–4.9) *

**N of cigarettes**: ≥ 20/day				
LIT (ref.)	7 (4/54)	1.0	2 (1/54)	1.0
HIT	13 (6/45)	1.9 (0.5–7.3)	13 (6/45) *	8.2 (0.9–70.5)
**N of cigarettes**: 10–19/day				
LIT (ref.)	19 (14/75)	1.0	12 (9/75)	1.0
HIT	24 (18/76)	1.4 (0.6–3.0)	17 (13/76)	1.5 (0.6–3.8)
**N of cigarettes**: 0–9/day				
LIT (ref.)	32 (6/19)	1.0	16 (3/19)	1.0
HIT	44 (11/25)	1.7 (0.5–5.9)	32 (8/25)	2.5 (0.6–11.2)

**Sex**: Male				
LIT (ref.)	12 (4/34)	1.0	6 (2/34)	1.0
HIT	23 (7/30)	2.3 (0.6–8.7)	17 (5/30)	3.2 (0.6–17.9)
**Sex**: Female				
LIT (ref.)	18 (20/114)	1.0	10 (11/114)	1.0
HIT	24 (28/116)	1.5 (0.8–2.8)	19 (22/116)	2.2 (1.01–4.8) *

*Note*: Tests of homogeneity of the ORs over education, intensity of smoking and sex, using the Breslow-Day test, showed that the null hypothesis of homogeneity could not be rejected for any of the variables.

* Statistically significant difference between the programs at the 5% level

^†^Point prevalence abstinence = not smoked at all in the seven days prior to follow-up. Continuous abstinence = not smoked at all in the 6 months (≥ 183 days) prior to follow-up.

In analyses of the 121 from the LIT-group and the 97 from the HIT-group who answered the complete follow-up questionnaire (not in table), we found that none of the people in the LIT-group who had abstained from seeking other support reported continuous abstinence, compared with 14% of those who had access to other support. Additional support had little (if any) additional effect on continuous abstinence in the HIT-group (quit rate 28% with additional support and 27% without). Point prevalence abstinence in the LIT-arm was 8% among those reporting to have had no access to other additional support and 23% among those reporting to have had access to other support. Corresponding point prevalence proportions in the HIT-group were 27% for those reporting no other support and 37% for those reporting to have had access to additional support. Regarding point prevalence abstinence, the OR (95% CI) for HIT compared to LIT was 4.3 (0.6–30.7) for participants without other support, and 2.0 (1.04–3.8) for participants with other support. For continuous abstinence, the OR (95% CI) for HIT compared to LIT was 2.5 (1.2–5.2) for participants with other support. However, for participants without other support it was impossible to compute OR due to too few individuals.

With the exception of point prevalence abstinence in the LIT-arm, people reporting periods of depressive mood during the previous 12 months were less likely to be abstinent at follow-up than people not reporting depressive mood, however, these differences were not significant in the present material (not in table).

142 of the 193 (74%) participants, responding to the questionnaires, who were still smoking at follow-up, intended to make a new quit attempt within the following 6 months, with no significant differences between the groups (not in table).

Half of the participants had used Nicotine Replacement Therapy (NRT) and there was no difference in NRT use between the LIT- and the HIT-group (not in table).

Twelve participants (6%) had used oral tobacco (snus) as a substitute for smoking after cessation, five in the LIT-group (three men and two women) and seven in the HIT-group (two men and five women) (not in table).

According to the multiple logistic regression analysis (Table 5), number of smoked cigarettes at baseline was the only variable with significant effect on the point prevalence abstinence, while type of program and number of smoked cigarettes (0–9/day compared to ≥ 20/day) were the only variables with significant effect on continuous abstinence, after controlling for sex, age and education level. For point prevalence abstinence, the controlled OR for being smoke-free was 5.5 when smoking 0–9 cigarettes/day compared to ≥ 20/day, and 2.4 when smoking 10–19 cigarettes/day compared to ≥ 20/day. For continuous abstinence, the controlled OR for HIT compared to LIT was 2.2 and for smoking 0–9 cigarettes/day compared to ≥ 20/day the controlled OR was 4.1. Sex and age had no effect on any of the outcome measures after control (not in table).

**Table 5:** Multivariate ORs and 95% CI for abstinence (point prevalence and continuous) controlled for sex, age, and other variables in the table.

		**Point prevalence abstinence**^†^	**Continuous abstinence**^†^
	
	% (n/N)	OR (95% CI)	OR (95% CI)
**Program**			
LIT (ref.)	50 (148/294)	1.0	1.0
HIT	50 (146/294)	1.5 (0.8–2.8)	2.2 (1.1–4.6) *

**Education**			
0–9 years (ref.)	23 (67/294)	1.0	1.0
≥ 10 years	77 (227/294)	1.7 (0.7–3.8)	1.6 (0.6–4.2)

**N of cigarettes**			
≥ 20/day (ref.)	34 (99/294)	1.0	1.0
10–19/day	51 (151/294)	2.4 (1.1–5.1) *	2.1 (0.9–5.3)
0–9/day	15 (44/294)	5.5 (2.2–13.5) ***	4.1 (1.5–11.8) **

* Statistically significant difference at the 5% level

** Statistically significant difference at the 1% level

*** Statistically significant difference at the 0.1% level

^† ^Point prevalence abstinence = not smoked at all in the seven days prior to follow-up. Continuous abstinence = not smoked at all in the 6 months (≥ 183 days) prior to follow-up.

In a drop-out (attrition) analysis we compared the baseline characteristics of participants answering the complete follow-up questionnaire (n = 218) with the participants who either did not respond to the follow-up questionnaire at all (n = 33) or the non-completers in the HIT-group who responded to the short follow-up questionnaire (n = 34). The drop-out group had higher cigarette consumption at baseline (p < 0.05), however no differences were seen between the groups regarding other variables presented in tables 1 and 2, nor snus or NRT use.

## Discussion

The HIT-protocol was significantly more effective than the LIT-protocol in terms of proportion of smokers reporting continuous abstinence at the 12-month follow-up.

### Treatment of choice

After controlling for sex, age, number of smoked cigarettes at baseline, and education level, the HIT-protocol was significantly more effective than the LIT-protocol for continuous abstinence, with OR of 2.2, but not for point prevalence abstinence. The relatively larger difference observed between the groups in the comparison using continuous abstinence as opposed to point prevalence abstinence is probably due to the nature of the different programs. The HIT program presumably gives participants more structure to keep program dates, which would increase the proportion of people quitting at a fixed date and consequently the proportion reporting to have been continuously abstinent for six months or longer at the time of follow-up. Most studies show point prevalence quit rates at 12-month follow-up between 7–10% for motivated smokers trying to quit without assistance [20]. Overall, the LIT-group was approximately twice as likely to report point prevalence abstinence. Thus, the LIT approach may be preferable as first treatment option since it is much less expensive and less time consuming. However, our data indicate that LIT participants should be encouraged to seek additional support. A formal cost effectiveness assessment comparing the different treatment protocols is presently being analysed and will be published in a separate paper.

### Other support

In the LIT-arm only 8% of those not having access to other additional support reported *point prevalence *abstinence. However, of the people in the LIT-group reporting to have had access to other support, 23% reported point prevalence abstinence at the 12-month follow-up, which is relatively high compared with spontaneous quit rates of motivated smokers trying to quit on their own [20]. Corresponding point prevalence proportions in the HIT-group were 27% for those reporting no other support and 37% for those reporting to have had access to additional support. None of the participants in the LIT-arm reported *continuous abstinence *in the absence of other support, in comparison to 14% of those reporting additional support. In the present study we only assessed if participants had sought other support (yes or no). Consequently we know neither the kind nor the intensity of the support.

The relatively high success rate of those LIT clients reporting additional support suggests the possibility of improving the effectiveness of the LIT intervention. One option may be telephone based quitlines [21,22].

### The self-help manual

One part of the LIT-program was the self-help manual. The value of such manuals for smoking cessation is under scrutiny [23]. The design of the present study does not allow us to isolate the possible effect of the self-help material, since the LIT-program was a combination of five factors comprising screening for tobacco use, offering support, one 30-minute treatment session, the 12-month follow-up, and the self-help material.

### Sex differences

The overwhelming majority of women in the present study reflects previous findings that Swedish women are more willing to seek and accept support for smoking cessation [16]. Also, where Swedish women tend to seek professional help and use medication to treat their nicotine dependency, Swedish men have tended to substitute smoking with other forms of tobacco, mainly the Swedish oral tobacco (snus). Consequently, approximately 32% of adult Swedish men are still daily tobacco users compared with approximately 18% of Swedish women [24].

### Other differences

Besides program, only number of smoked cigarettes at baseline had a significant influence on the probability of abstinence, controlled for the other variables. In accordance with previously reported data, people reporting depressive mood after the quit date were less likely to report abstinence at the follow-up [16], with the exception of point prevalence abstinence in the LIT-arm. The lack of statistical significance in the present analysis is probably due to insufficient statistical power. It should be noted that depressive mood does not refer to clinical depression. People with lower levels of education appeared to gain more from the HIT-protocol with more repeated support.

There was no difference in education level between those 43 HIT participants who did not complete the program and those who did.

### Specific methodological problems

A problem in the present study is the lack of detailed follow-up information from those 43 people in the HIT-arm who did not complete the program. Although this does not affect the main outcome measure since all participants received the three central questions regarding abstinence, it creates problems in the comparison between the arms of other variables included in the more extensive questionnaire, such as other support and depressive mood. The decision to retrieve only the most relevant information from those "dropping out" of the HIT-arm was based on the belief that the response rate from these people would be low if they received the longer version of the questionnaire. The few individuals with lower education in HIT and LIT respectively, as seen in Table 4, may also be a problem regarding the possibility to find statistically significant differences between the programs. However, this fact indicates that Sweden has a rather well-educated population.

We did not attempt to retrieve information from the LIT-group regarding to what extent they attempted to follow the program manual. We argued that the potential recall bias introduced by collecting such data retrospectively almost one year later would make the findings highly unreliable. An alternative way would have been to collect the data prospectively. However, introducing that level of proactivity into the LIT-arm would significantly have enhanced the treatment intensity of the LIT program.

A possible weakness of the present study may be the lack of chemical validation of abstinence. However, since participants were free to use oral tobacco and NRT, it would be problematic to distinguish low levels of smoking and use of oral tobacco or NRT. Furthermore, if people tended to lie about their smoking behaviour we would expect a similar distribution of untruthful answers in both arms of this randomized study.

## Conclusion

The results of the present study indicate that screening for willingness to quit smoking within the health care system and offering smoking cessation support within dentistry may be an effective model for smoking cessation support in Sweden. The LIT-approach is probably less expensive and time consuming per quitter and may be a preferable "first treatment option". However, it should be integrated with other kinds of available support. The more extensive and expensive HIT-protocol is more effective in terms of proportion of smokers who are smoke-free after 12 months and should be offered to those who are unable to quit with LIT-support in combination with other support.

## Competing interests

The authors declare that they have no competing interests.

## Authors' contributions

EN: data collection and analysis, manuscript preparation, (project co-ordinator), ÅT: study design, manuscript preparation, (project leader), PT: study design, construction and validation of outcome measures, manuscript preparation, PJ: construction of outcome measures, AR: statistical analysis, consultation, manuscript preparation, ARH: study design, construction and validation of outcome measures, data analysis, manuscript preparation. All authors read and approved the final manuscript.

## Pre-publication history

The pre-publication history for this paper can be accessed here:


